# Paricalcitol Ameliorates Acute Kidney Injury in Mice by Suppressing Oxidative Stress and Inflammation via Nrf2/HO-1 Signaling

**DOI:** 10.3390/ijms24020969

**Published:** 2023-01-04

**Authors:** Shuang Wang, Siqi Huang, Xingyao Liu, Yanjun He, Yun Liu

**Affiliations:** 1 College of Veterinary Medicine, Northeast Agricultural University, Harbin 150030, China; 2 Heilongjiang Province Key Laboratory of Pathogenic Mechanism for Animal Disease and Comparative Medicine, Harbin 150030, China

**Keywords:** acute kidney injury, paricalcitol, oxidative stress, inflammation, *Nrf2*/*HO-1* signaling

## Abstract

Effective and targeted prevention and treatment methods for acute kidney injury (AKI), a common clinical complication, still needs to be explored. Paricalcitol is a biologically active chemical that binds to vitamin D receptors in the body to exert anti-oxidant and anti-inflammatory effects. However, the molecular mechanism of the effect of paricalcitol on AKI remains unclear. The current study uses a paricalcitol pretreatment with a mouse AKI model induced by cisplatin to detect changes in renal function, pathology and ultrastructure. Results showed that paricalcitol significantly improved renal function in mice and reduced inflammatory cell infiltration and mitochondrial damage in renal tissue. Furthermore, paricalcitol markedly suppressed reactive oxygen species and malondialdehyde levels in the kidneys of AKI mice and increased the levels of glutathione, superoxide dismutase, Catalase and total anti-oxidant capacity. In addition, we detected renal necrosis and inflammation-related proteins in AKI mice by immunofluorescence and Western blot, and found that their levels were markedly decreased after paricalcitol pretreatment. Moreover, paricalcitol promotes nuclear factor erythroid 2-related factor 2 (*Nrf2*) in the nucleus and activates the *Nrf2*/heme oxygenase-1 (*HO-1*) signaling pathway; while HO-1 is inhibited, the protective effect of paricalcitol on the kidney is attenuated. In conclusion, paricalcitol exerts a renoprotective effect by decreasing renal oxidative injury and inflammation through *Nrf2*/*HO-1* signaling, providing a new insight into AKI prevention.

## 1. Introduction

Acute kidney injury (AKI) is a common clinical syndrome characterized by a sharp decline in renal function over a short period of time. The pathogenesis of AKI is complex and multiple pathological processes are involved, such as inflammation, apoptosis, necrosis, etc. [1,2,3]. Clinically, major surgery, radiocontrast agent exposure and drug nephrotoxicity can all induce AKI. A large-scale multicenter epidemiological survey of critically ill patients showed that AKI induced by drug nephrotoxicity accounted for 19% [4] of cases. Moreover, AKI treatment costs are high, and it is likely to develop into chronic kidney disease, which affects the patients’ quality of life not only economically but also healthwise. To make matters worse, the incidence of AKI is still rising worldwide. Cisplatin (CDDP), an effective anti-solid tumor drug, is one of the main causes of AKI in clinical practice. However, the current prevention and treatment methods for AKI are very limited; the protection of kidneys in chemotherapy patients is an especially urgent problem that needs to be solved. Thus, it is very necessary to explore effective methods to prevent AKI.

Necroptosis is a cell death mode controlled by receptor interacting protein (RIP) kinases, which can lead to the disruption of the cell membrane, resulting in the leakage of cellular contents and an inflammatory response in the surrounding tissues [5,6]. Notably, necroptosis is not dependent on the proteolytic activity of cysteinyl aspartate specific proteinase; in contrast, only when cysteinyl aspartate specific proteinase 8 (*Cas8*) is inactivated, *RIP1* and *RIP3* can phosphorylate each other to form necrosomes. A previous study showed that the expression of RIP1, RIP3 and inflammatory factors such as tumor necrosis factor-α (*TNF-α*) increased in a cisplatin-induced mouse model of tubular injury, while the inhibition of *RIP1* or *RIP3* improved tubular injury and reduced the upregulation of inflammatory factors [7]. Besides, nuclear factor kappa B (*NF-κB*), a nuclear transcription factor, plays a non-negligible role in the process of inducting AKI by mediating inflammatory factors including *TNF-α* and interleukin-1β (*IL-1β*) [8]. Furthermore, it has been reported that *RIP3* is significantly elevated in mouse lupus nephritis and activates the renal necroptosis pathway, whereas the administration of an *RIP3* inhibitor to mice obviously attenuates lupus nephritis [9]. More and more studies have shown that necroptosis is often accompanied by the accumulation of reactive oxygen species (ROS), and a large amount of ROS can easily induce oxidative stress [10]. Oxidative stress is also one of the triggers of AKI [11]―that is, anti-oxidant substances such as superoxide dismutase (SOD), glutathione (GSH) and glutathione peroxidase 4 (GPX4) in the renal tissue cannot remove oxidative products such as ROS and malondialdehyde (MDA), and the dynamic imbalance between renal oxidation and anti-oxidants leads to AKI. Therefore, blocking necroptosis-mediated inflammation and oxidative stress is an effective way to improve AKI.

Nuclear factor erythroid 2-related factor 2 (*Nrf2*), an important transcription factor, is anchored in the cytoplasm by Kelch-like ECH-associated protein 1 (*Keap1*) under normal physiological conditions; once cells receive adverse stimuli such as ROS, it will dissociate from *Keap1* and enter the nucleus to regulate downstream gene expression and activate anti-oxidant enzymes, thereby resisting intracellular oxidative stress [12,13]. Heme oxygenase-1 (HO-1) is not only an anti-oxidant protein regulated by Nrf2, but is also known as an inducible heat shock protein 32, whose level is elevated under external stimuli such as oxidative stress and inflammation [14,15]. Studies have found that tanshinone I exerts cardiovascular protection by inhibiting *RIP1*/*RIP3* and thereby reducing necroptosis, which is associated with the upregulation of *Nrf2* and *HO-1* [16]. Furthermore, Nrf2/HO-1 signaling is involved in the regulation of inflammation. It is reported that gallic acid effectively mitigates lung inflammation and oxidative stress by activating *Nrf2*/*HO-1* signaling [17]. Wang et al. found that the transfer of *Nrf2* into the nucleus to activate anti-oxidant enzymes including HO-1 is the key for aucubin to alleviate encephalitis, while the knockout of *Nrf2* weakens the anti-inflammatory effect of aucubin [18]. In addition, *Nrf2*/*HO-1* signaling has a crucial role in different kidney diseases such as diabetic nephropathy, ischemia-reperfusion kidney injury and chronic kidney disease [19,20,21]. Hence, the upregulation of *Nrf2*/*HO-1* signaling in renal tissue appears to be beneficial for ameliorating AKI.

Paricalcitol is a biologically active synthetic chemical that binds to intracellular vitamin D receptors to exert biological effects. A recent study has shown that paricalcitol restrains the expression of pro-inflammatory factors caused by the translocation of *NF-κB* from cytoplasm to nucleus, thereby improving experimental autoimmune encephalomyelitis [22]. It has also been found that paricalcitol reduces liver damage caused by bile duct ligation through suppressing oxidative stress [23]. Clinically, paricalcitol has been used to treat secondary hyperparathyroidism in patients with chronic kidney injury [24]. Moreover, a recent study found that paricalcitol ameliorated experimental chronic renal failure-induced renal interstitial fibrosis by altering the renin and angiotensin receptors and reducing renal inflammation [25]. However, the effects of paricalcitol on AKI and related mechanisms are still rarely reported. Given this, in this experiment, we explored the effect of paricalcitol on cisplatin-induced AKI from oxidative stress and inflammation, using *HO-1* inhibitors to determine whether paricalcitol exerts nephroprotective effects through the *Nrf2*/*HO-1* signaling pathway. These findings not only provide valuable data for paricalcitol as a renal protective agent, but also provide new clues for the prevention of AKI.

## 2. Results

### 2.1. The Effect of Paricalcitol on Cisplatin-Induced AKI

**Figure 1:**
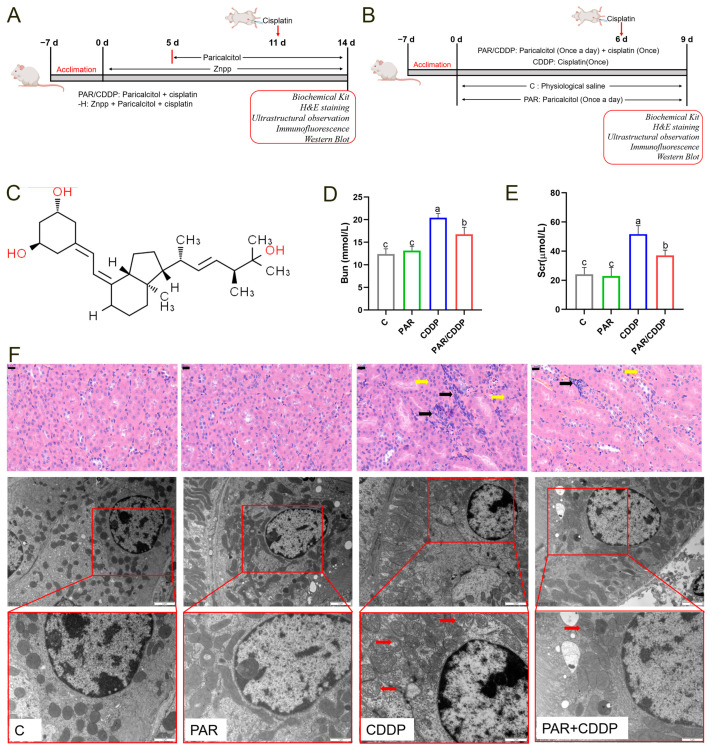
The effect of paricalcitol on cisplatin-induced AKI. (**A**,**B**) Schematic of the experiment. (**C**) Chemical structure formula of paricalcitol. (**D**) BUN level in mice blood (*n* = 6). (**E**) SCr level in mice serum (*n* = 6). (**F**) Histopathological examination (scale bars, 20 μm) and transmission electron microscopic images (scale bars, 2 μm and 1 μm) of mice kidneys (*n* = 3). Yellow arrow, renal tubule epithelium cells injury; Black arrow, inflammatory cell infiltration; Red arrow, mitochondrial damage. C, control group; PAR, paricalcitol group; CDDP, cisplatin group; PAR/CDDP, paricalcitol + cisplatin group. Bars that do not share the same letters are significantly different (*p* < 0.05) from each other. Results are presented as Mean ± SD. Statistical significance was obtained by one-way ANOVA.

To explore the effect of paricalcitol activation on a mouse kidney, we determined the levels of BUN and SCr in mouse blood and serum. As shown in Figure 1, the levels of BUN and SCr were significantly increased in the mice treated with cisplatin, whereas paricalcitol distinctly reduced these alterations (*p* < 0.05) (Figure 1D,E). Histopathological examination revealed that in the CDDP group, the renal tubular epithelial cells were damaged (yellow arrow) and many inflammatory cells were infiltrated (black arrow) compared with those in the C group. By contrast, paricalcitol partially ameliorated these abnormal changes (Figure 1F). Furthermore, the TEM results indicated that there was cisplatin-induced mitochondrial damage in the renal tissue of AKI mice, manifested as mitochondrial cristae fragmentation (red arrow), whereas paricalcitol markedly decreased this alteration (Figure 1F).

### 2.2. Effect of Paricalcitol on Oxidation Products in Mice Kidneys

**Figure 2:**
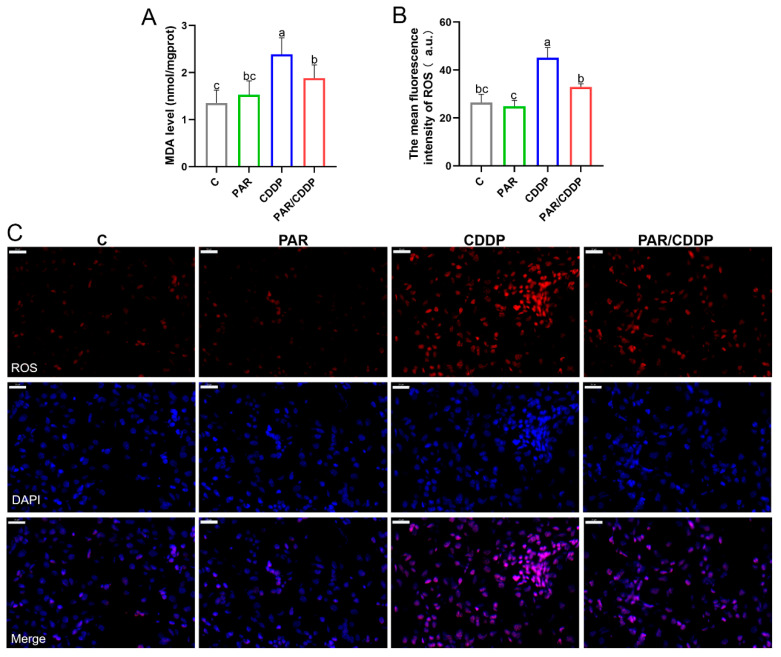
Effect of paricalcitol on oxidation products in mice kidneys. (**A**) The content of MDA in mice kidneys (*n* = 6). (**B**,**C**) Representative images and quantification of ROS (red) detection by fluorescence microscopy (scale bars, 20 μm; *n* = 3). C, control group; PAR, paricalcitol group; CDDP, cisplatin group; PAR/CDDP, paricalcitol + cisplatin group. Bars that do not share the same letters are significantly different (*p* < 0.05) from each other. Results are presented as Mean ± SD. Statistical significance was obtained by one-way ANOVA.

In order to explore whether paricalcitol affects the accumulation of renal oxidation products, the levels of ROS and lipid peroxidation product MDA in mice kidneys were detected. As shown in Figure 2, the content of MDA in the CDDP group was significantly increased compared to the C group, whereas paricalcitol markedly restored this change (*p* < 0.05) (Figure 2A). Besides, compared with the C group, the accumulation of ROS in the kidneys of AKI mice was significantly increased. The level of ROS in the PAR/CDDP group was obviously lower than that in the CP group (Figure 2B,C) (*p* < 0.05).

### 2.3. Effect of Paricalcitol on Anti-oxidant Level of Mice Kidneys

**Figure 3:**
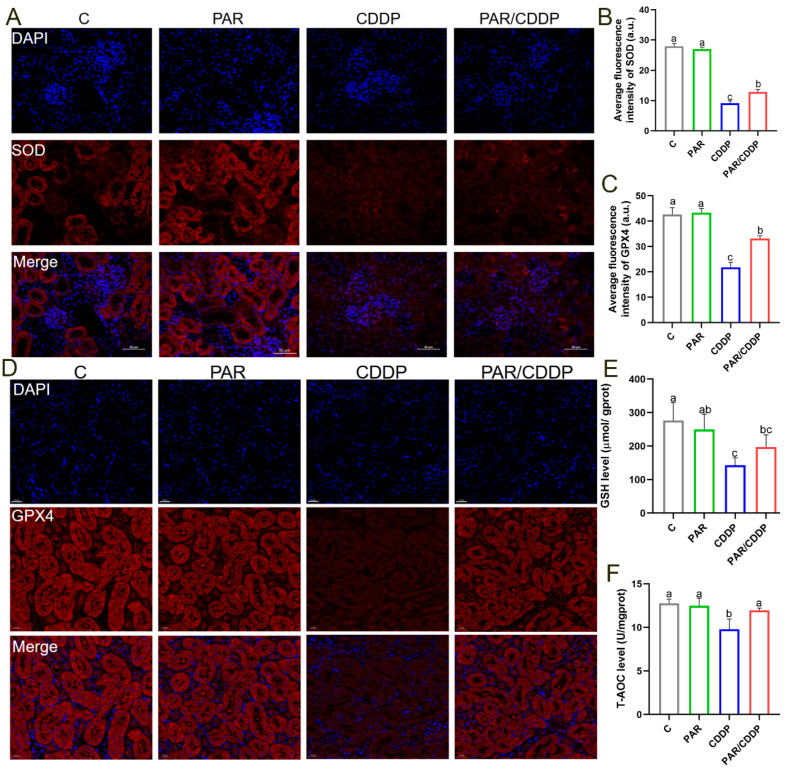
Effect of paricalcitol on anti-oxidant level of mice kidneys. (**A**,**B**) Representative images and quantification of SOD (red) detection by fluorescence microscopy (scale bars, 50 μm; *n* = 3). (**C**,**D**) Representative images and quantification of GPX4 (red) detection by fluorescence microscopy (scale bars, 20 μm; *n* = 3). (**E**,**F**) The levels of GSH and T-AOC in mice kidneys (*n* = 6). C, control group; PAR, paricalcitol group; CDDP, cisplatin group; PAR/CDDP, paricalcitol + cisplatin group. Bars that do not share the same letters are significantly different (*p* < 0.05) from each other. Results are presented as Mean ± SD. Statistical significance was obtained by one-way ANOVA.

To investigate the effect of paricalcitol on anti-oxidant levels in renal tissue, we determined the levels of SOD and GPX4 by immunofluorescence and observed that paricalcitol markedly enhanced their levels in the renal tissues of the AKI mice (Figure 3A–D) (*p* < 0.05). Additionally, the GSH content and T-AOC level in the CDDP group were significantly decreased compared to the C group, while paricalcitol markedly improved these changes (Figure 3E,F) (*p* < 0.05). These findings suggest that paricalcitol prominently enhances the anti-oxidant capacity of the kidney.

### 2.4. Paricalcitol Reduced Necroptosis and Inflammation in Cisplatin-Treated Mice Kidneys

**Figure 4:**
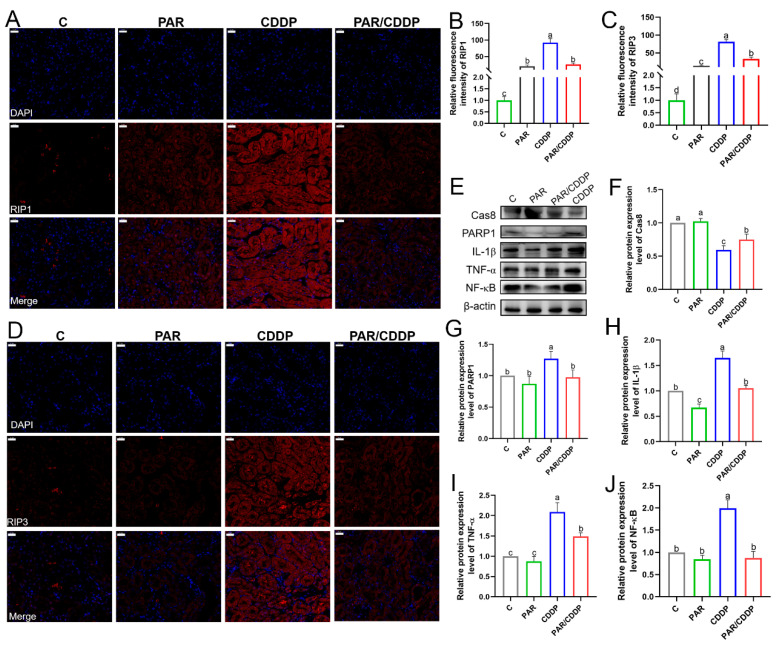
Paricalcitol reduced necroptosis and inflammation in cisplatin-treated mice kidneys. (**A**–**D**) Immunofluorescence representative images and quantification of RIP1 and RIP3 (scale bars, 20 μm; *n* = 3). (**E**–**J**) Western blotting for Cas8, PARP1, IL-1β, TNF-α and NF-κB in the mice kidneys (*n* = 3). C, control group; PAR, paricalcitol group; CDDP, cisplatin group; PAR/CDDP, paricalcitol + cisplatin group. Bars that do not share the same letters are significantly different (*p* < 0.05) from each other. Results are presented as Mean ± SD. Statistical significance was obtained by one-way ANOVA.

The expression of necroptosis-related and inflammation-related genes were also determined. As shown in Figure 4, a significant increase in the levels of RIP1 and RIP3 in the CDDP group compared with those in the C group was observed, whereas paricalcitol was found to mitigate this variation (Figure 4A–D) (*p* < 0.05). Compared with those in the CDDP group, the protein level of polymerase-1 (PARP1) significantly decreased and that of Cas8 was markedly enhanced in the other three groups (Figure 4F,G) (*p* < 0.05). Furthermore, paricalcitol pretreatment significantly reduced the protein levels of pro-inflammatory cytokines, such as IL-1β, TNF-α and NF-κB, in the kidneys of AKI mice (Figure 4G–J) (*p* < 0.05).

### 2.5. Paricalcitol Activated the Nrf2/HO-1 Signaling Pathway in Mice Kidneys

**Figure 5:**
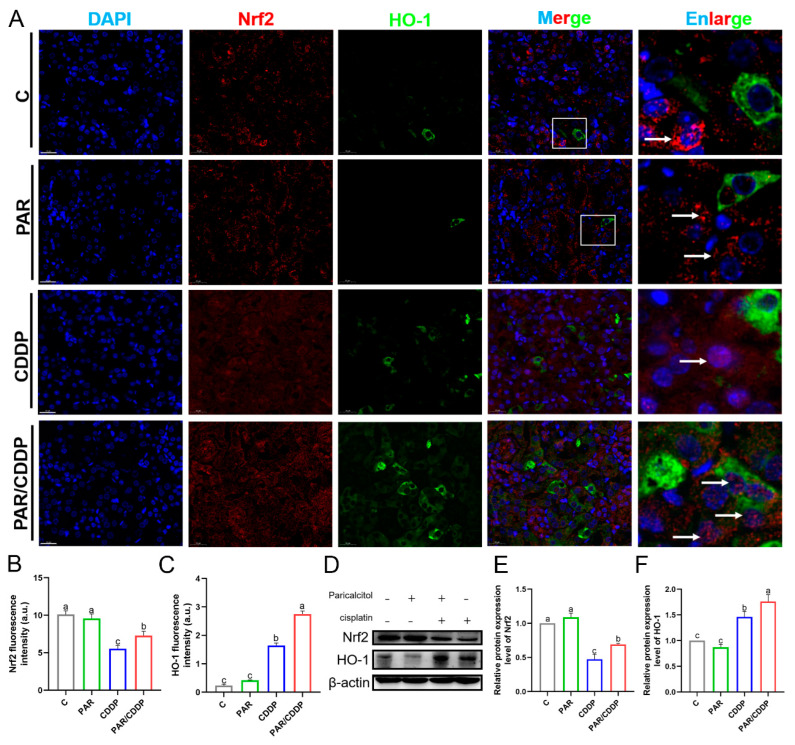
Paricalcitol activated the Nrf2/HO-1 signaling pathway in mice kidneys. (**A**–**C**) Representative images and quantification for immunofluorescence of Nrf2 (red) and HO-1 (green) (scale bars, 20 μm; *n* = 3). (**D**–**F**) The protein expressions of Nrf2 and HO-1 in mice kidneys (*n* = 3). C, control group; PAR, paricalcitol group; CDDP, cisplatin group; PAR/CDDP, paricalcitol + cisplatin group. Bars that do not share the same letters are significantly different (*p* < 0.05) from each other. Results are presented as Mean ± SD. Statistical significance was obtained by one-way ANOVA.

To explore the changes in *Nrf2*/*HO-1* signaling with paricalcitol alleviating cisplatin-induced AKI, we detected the levels of Nrf2 and HO-1 by immunofluorescence double staining. As shown in Figure 5, Nrf2 (red) mainly existed in the cytoplasm in the C group and the PAR group, whereas numerous Nrf2 was observed to enter the nucleus obviously in the PAR/CDDP group (white arrow) (Figure 5A). Moreover, Nrf2 was also present in a small number of nuclei in the kidneys of AKI mice (Figure 5A). Quantification of the mean fluorescence intensity of Nrf2 (red) and HO-1 (green) found that paricalcitol significantly enhanced their levels in the kidneys of AKI mice (Figure 5B,C) (*p* < 0.05). Compared with the C group, the average fluorescence intensity of Nrf2 weakened and the average fluorescence intensity of HO-1 increased in the kidneys of mice in the CDDP group (Figure 5B,C) (*p* < 0.05). The protein expressions of Nrf2 and HO-1 detected by western blot are consistent with the results of fluorescent staining (Figure 5D–F) (*p* < 0.05).

### 2.6. Inhibiting HO-1 Expression Reduces the Anti-Oxidation Levels of Paricalcitol

**Figure 6:**
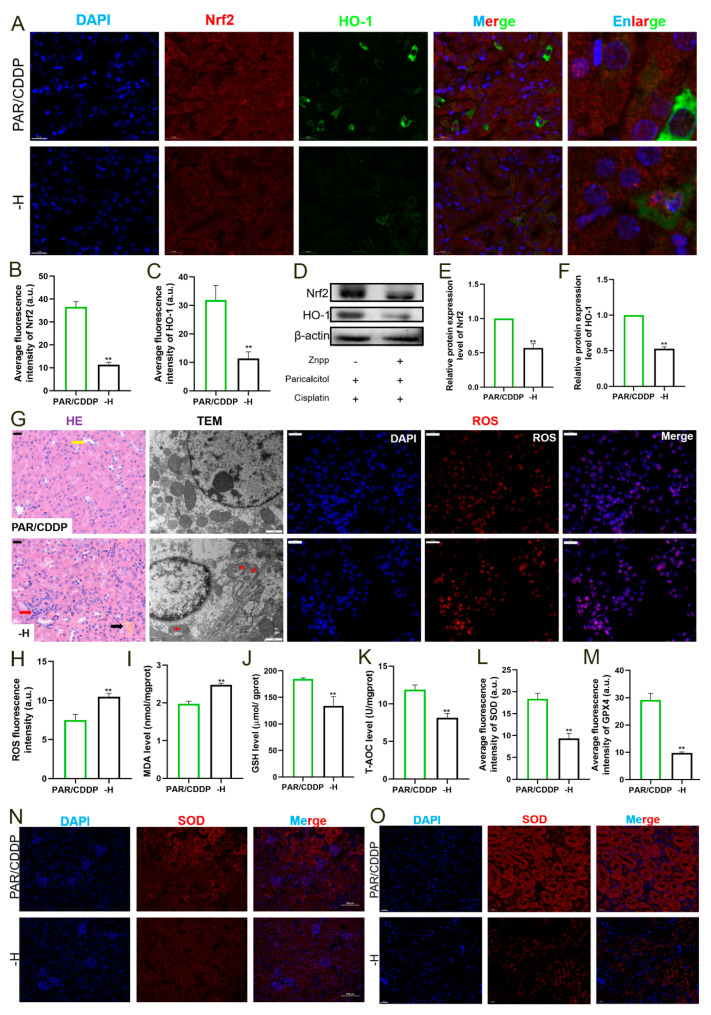
Inhibiting HO-1 expression reduces the anti-oxidation levels of paricalcitol. (**A**–**C**) Representative images and quantification for immunofluorescence of Nrf2 (red) and HO-1 (green) (scale bars, 20 μm; *n* = 3). (**D**–**F**) The protein expressions of Nrf2 and HO-1 in mice kidneys (*n* = 3). (**G**) Representative images of HE (scale bars, 20 μm), TEM (scale bars, 1 μm) and ROS (scale bars, 20 μm) in mice renal tissues (*n* = 3). (**H**) ROS quantification. (**I**–**K**) The levels of MDA, GSH and T-AOC in mice kidneys (*n* = 6). (**L**–**O**) Representative images and quantification for immunofluorescence of SOD and GPX4 (scale bars, 20 μm; *n* = 3). PAR/CDDP, paricalcitol + cisplatin group; -H, ZnPP+ paricalcitol + cisplatin group. ** *p* < 0.01. Dates are presented as Mean ± SD. Statistical significance was obtained by unpaired Student’s *t*-test.

To further determine whether paricalcitol exerts renal protection through the *Nrf2*/*HO-1* signaling pathway, we used paricalcitol to pretreat AKI mice with *HO-1* inhibited and unsurprisingly found that the renoprotective effect of paricalcitol was diminished. The levels of Nrf2/HO-1 in the -H group were significantly decreased compared with those in the PAR/CDDP group (Figure 6A–F) (*p* < 0.05). Moreover, compared with the PAR/CDDP group mice, those in the -H group had more severe renal tissue damage, which manifested as massive inflammatory cell infiltration (red arrow), mitochondrial vacuolization (red pentagram) and increased ROS accumulation (Figure 6A,B). In addition, the content of MDA in the -H group was significantly increased and the levels of GSH, T-AOC, SOD and GPX4 were markedly enhanced compared to those in the PAR/CDDP group (Figure 6I–O) (*p* < 0.01).

### 2.7. Inhibiting HO-1 Expression Reduces the Anti-Inflammatory Levels of Paricalcitol

**Figure 7:**
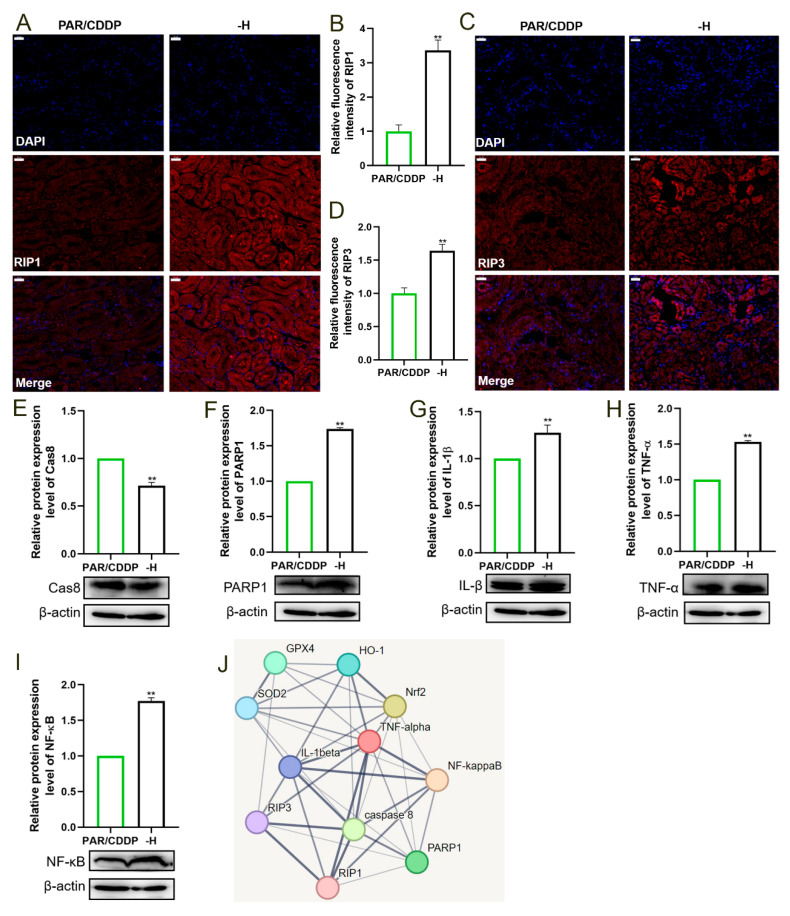
Inhibiting HO-1 expression reduces the anti-inflammatory levels of paricalcitol. (**A**,**B**) Immunofluorescence representative images and quantification of RIP1 (scale bars, 20 μm; *n* = 3). (**C**,**D**) Immunofluorescence representative images and quantification of RIP3 (scale bars, 20 μm; *n* = 3). (**E**–**I**) The protein expressions of Cas8, PARP1, IL-1β, TNF-α and NF-κB (*n* = 3). (**J**) Interaction diagram of related proteins in this experiment. PAR/CDDP, paricalcitol + cisplatin group; -H, ZnPP+ paricalcitol + cisplatin group. ** *p* < 0.01. Dates are presented as Mean ± SD. Statistical significance was obtained by unpaired Student’s *t*-test.

To investigate whether the inhibition of *Nrf2*/*HO-1* signaling had an effect on paricalcitol in reducing renal inflammation induced by necroptosis, we detected the expressions of key genes regulating necroptosis and inflammation. As shown in Figure 7, the protein levels of RIP1 and RIP3 distinctly increased and the Cas8 level significantly decreased in the -H group compared with those in the PAR/CDDP group (Figure 7A–E) (*p* < 0.01). Furthermore, compared with the PAR/CDDP group, the protein expressions of PARP1, IL-1β, TNF-α and NF-κB were markedly increased in the -H group (Figure 7F–I) (*p* < 0.01). The interaction between each protein is shown in the Figure 7J.

## 3. Discussion

**Figure 8:**
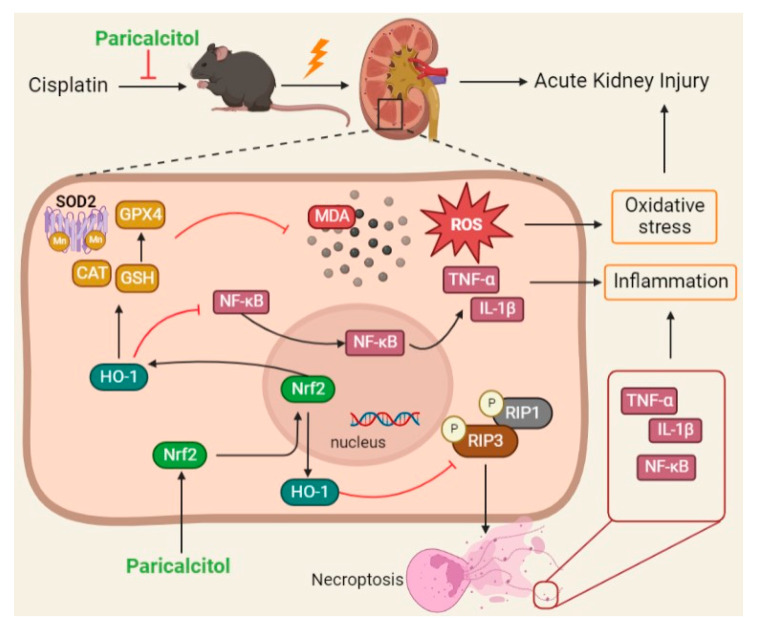
The schematic diagram of paricalcitol upregulating Nrf2/HO-1 signaling to improve cisplatin-induced kidney injury. Cisplatin induces oxidative stress and inflammation by promoting the accumulation of oxidative products (ROS and MDA) and the secretion of inflammatory factors (IL-1β, TNF-α and NF-κB). Paricalcitol plays a protective role in the kidney by enhancing renal anti-oxidant and anti-inflammatory levels via Nrf2/HO-1 signaling.

AKI is a clinical syndrome characterized by the rapid decline of renal function, and there are currently no very effective prevention and treatment methods. Cisplatin is one of the most widely used antitumor drugs, and its side effects on kidneys limit its clinical application. A recent prospective study reported that 25-hydroxyvitamin D deficiency increases the risk of hospitalization due to AKI [26], suggesting that paricalcitol, as a chemical related to vitamin D function, has a positive effect on AKI. In the current study, paricalcitol was used to pretreat cisplatin-induced AKI mice to observe pathological changes in renal tissues and detect renal function and related gene expressions. Our results showed that paricalcitol activation upregulated the *Nrf2*/*HO-1* signaling pathway in the renal tissues of AKI mice and significantly reduced inflammation and oxidative stress. However, the renoprotective effect of paricalcitol was weakened considerably in HO-1 inhibited AKI mice, further suggesting that paricalcitol protects the kidney through the *Nrf2*/*HO-1* signaling pathway (Figure 8).

All biological systems exist in redox equilibrium. Once this balance is broken, the oxidative substances in the body cannot be removed in time and eventually lead to oxidative stress [27]. Moreover, an excessive production of ROS in the body will oxidize the biofilm and induce lipid peroxidation [28]. MDA is one of the main products of lipid peroxidation, which can be scavenged by anti-oxidant substances such as SOD and GSH. It was reported that the content of the oxidation product MDA in 10 patients with sepsis-associated AKI was significantly higher than that in healthy patients [29]. In addition, studies have shown that oxidative damage can aggravate cisplatin-induced AKI, whereas scavenging oxygen free radicals can effectively protect against AKI [30,31]. Thus, reducing renal oxidative stress is an effective way to improve AKI. In this present study, we found that the oxidizing substances in the renal tissue of AKI mice were significantly decreased after paricalcitol pretreatment, while the renal anti-oxidant system was activated. In detail, paricalcitol enhanced the activity of anti-oxidant enzymes SOD and GPX4, increased the level of anti-oxidant GSH and the total anti-oxidant capacity, and then improved the ability to scavenge ROS and MDA to protect the kidney from oxidative stress. Furthermore, the inflammatory response of renal tissues in cisplatin-induced AKI is well-documented [32]. *NF-κB* in the kidney is activated via its translocation to the nucleus after cisplatin stimulation, which then promotes the transcription of inflammatory mediators including TNF-α; TNF-α, pharmacologically inhibited, would decrease the expression of pro-inflammatory factors such as *IL-1β* [33,34,35]. In addition, *PARP-1* regulates *TNF-α* and *IL-1β* expression, and inhibition of *PARP-1* abates cisplatin-induced inflammation in renal tissues. Our results showed that inflammatory factors in renal tissues increased in cisplatin-induced AKI mice, whereas paricalcitol significantly reversed this change, indicating that paricalcitol can decrease the renal inflammatory response. Moreover, *TNF-α* can bind to tumor necrosis factor receptors on the cell membrane and promote the formation of necrosomes by *RIP1*/*RIP3* in the case of *Cas8* inactivation, thereby inducing necroptosis [36]. Necroptotic cells release inflammatory cytokines after membrane disruption [37]. In the study by Chen et al., *RIP3* deficiency significantly suppressed necroptotic cells to secrete the inflammatory factor *IL-1β*, whereas *RIP3*/*MLKL*-dependent necroptosis triggered necro-inflammation, inducing the progression of AKI into chronic kidney disease [38]. Thus, preventing the necroptosis of renal cells can benefit patients with AKI. Our results showed that paricalcitol markedly decreased the level of RIP1 and RIP3 in the renal tissues of AKI mice and increased the activity of Cas8, indicating that paricalcitol exerts a beneficial effect on the kidney by reducing necroptosis.

*Nrf2*, as a transcription factor, can regulate the corresponding downstream genes and anti-oxidant enzymes after entering the nucleus. HO-1 is not only regulated by Nrf2 to play an anti-oxidant role, but can also be highly expressed by oxidative stress induced by various factors such as H_2_O_2_, heavy metals, endotoxin, etc. At present, *Nrf2*/*HO-1* signaling has been found to have anti-oxidative and anti-inflammatory effects in organisms. For instance, a previous study has shown that isoliquiritigenin upregulates *Nrf2*/*HO-1* signaling to prevent oxidative stress and inflammation, thereby alleviating caerulein-induced mild acute pancreatitis [39]. Moreover, many studies have shown that the activation of the *Nrf2*/*HO-1* signaling pathway has a positive effect on a variety of renal diseases [40,41]. However, Tang et al. found that sodium iodate-induced retinal pigment epithelium degeneration is associated with ferroptosis mediated by the upregulation of *Nrf2*/*SLC7A11*/*HO-1* signaling [42]. Another study has shown that ferroptosis aggravated renal tubular damage in a mode of diabetic nephropathy, which was related to the level of HO-1 [43]. Therefore, it is necessary to explore the role of *Nrf2*/*HO-1* signaling in the process of paricalcitol exerting renal protection. Here, we found that after paricalcitol treatment, the levels of Nrf2 and HO-1 in the mice kidneys were significantly increased, suggesting that paricalcitol exerts a reno-protective effect by upregulating *Nrf2*/*HO-1* signaling. Notably, we found that *Nrf2* entered the renal nucleus after cisplatin stimulation, and the protein expression of HO-1 was significantly increased accordingly, indicating that HO-1 may be regulated by Nrf2 or, as a stress protein, upregulated due to nephrotoxic cisplatin stimulation. Although the expression of HO-1, which has anti-oxidant effects, was increased in the kidneys of AKI mice, it did not prevent the occurrence of AKI, indicating that renal self-regulation and protection are limited. In addition, in order to further identify the role of the *Nrf2*/*HO-1* signaling pathway in the process of paricalcitol protecting the kidneys, we use ZnPP to inhibit *Nrf2*/*HO-1* signaling according to previous reports [44] and found that, unsurprisingly, the positive effect of paricalcitol on the renal tissues of AKI mice was attenuated.

## 4. Material and Methods

### 4.1. Animal Experimental Design

The animals used in this experiment were approved by the Institutional Animal Care and Use Committee of Northeast Agricultural University (SRM-11). All male *C57BL/6* mice (7-week-old) in this experiment were purchased from Changsheng Biotechnology Company (Changchun, China) and all of them were fed under SPF condition. The mice were acclimatized to natural light/dark cycles at a controlled temperature of 22 ± 2 °C with free access to food and water. The experiment is divided into two parts. Study 1: The mice were assigned to four groups: the control group (C group; *n* = 6), paricalcitol group (PAR group; *n* = 6), cisplatin group (CDDP group; *n* = 6) and paricalcitol + cisplatin group (PAR + CDDP group; *n* = 6). Paricalcitol and cisplatin were purchased from Dalian Meilun Biological Co., Ltd. (Dalian, China) and Sigma–Aldrich Co. (St. Louis, MI, USA), respectively. Paricalcitol (0.2 μg/kg) was injected once daily for nine days and cisplatin (20 mg/kg) was injected once on the sixth day [45]. The schematic of animal study 1 is shown in Figure 1A. Study 2: The mice were assigned to two group: the paricalcitol + cisplatin group (PAR + CDDP group; *n* = 6) and zinc protoporphyrin IX (ZnPP, HO-1 inhibitor) + paricalcitol + cisplatin group (-H group; *n* = 6). ZnPP was purchased from Dalian Meilun Biological Co., Ltd. (China). ZnPP (5 mg/kg) was injected five days before the mice were given paricalcitol until the end of the experimental modeling. The schematic of animal study 2 is shown in Figure 1B. The dosage and formulation methods of all drugs are based on previous reports [45,46]. After 72 h of cisplatin treatment, all animals were anesthetized using 5% isoflurane to collect the blood and then euthanized to collect renal tissues. For microscopic observation, a portion of the tissues were fixed in 10% paraformaldehyde or 2.5% glutaraldehyde phosphate. The remaining kidney tissues were rapidly quenched in liquid nitrogen and then stored at −80 °C for subsequent experiments.

### 4.2. Biochemical Kit

The levels of blood urea nitrogen (BUN), serum creatinine (SCr), MDA, total anti-oxidant capacity (T-AOC) and GSH were evaluated using a biochemical kit purchased from Nanjing Jiancheng, Co., Ltd. (Nanjing, China). All steps were carried out according to the kit instructions.

### 4.3. Hematoxylin–Eosin Staining

The mice kidney tissues were rapidly fixed in 10% formaldehyde for at least 24 h and then embedded in paraffin for microscopic examination. For the paraffin block, sections (5-μm thick) were cut and stained with hematoxylin and eosin (H&E) for light microscopic observation.

### 4.4. Transmission Electron Microscope

Small pieces from the kidney tissues of the C57BL/6J mice were fixed in 2.5% glutaraldehyde in 0.1 M phosphate buffer (pH 7.4) for 2 h at room temperature. After rinsing in 0.1 M phosphate buffer, specimens were post-fixed with 1% osmium tetroxide for 2 h, dehydrated in a graded series of ethanol, embedded in acetone and sectioned at approximately 70 nm. Ultrathin sections were stained with 3% uranyl acetate and lead citrate and viewed under a transmission electron microscope (TEM).

### 4.5. ROS Detection

Cryosections from frozen kidney tissues (5 μm) were prepared using a Leica CM1900 cryostat (Leica). As previously described [47], the sections were stained with dihydroethidium (DHE) solution for 30 min in the dark at 37 °C and then washed three times with phosphate-buffered saline (PBS). Finally, a fluorescence microscope (Olympus; Tokyo, Japan) was used to photograph the section.

### 4.6. Immunofluorescence

Renal tissues were fixed in 4% paraformaldehyde for 48 h, embedded in paraffin and sliced into 5 μm sections based on routine protocols. Immunofluorescence (IF) was performed as previously described [48]. The fluorescence intensities of GPX4 (ABclonal; 1:50), SOD (Bioss; 1:100), RIP1 (Bioss; 1:100), RIP3 (Bioss; 1:100), Nrf2 (Bioss; 1:100) and HO-1 (Proteintech; 1:50) in the photos were detected by ImageJ2 software.

### 4.7. Western Blot

Total protein extraction was performed using the Whole Cell Lysis Assay (Wanlei Biological Co., Ltd., Shenyang, China) according to the manufacturer’s protocol. Western blotting was performed, as previously described [49]. Briefly, protein samples were separated using SDS-PAGE gels and transferred to the PVDF membrane and combined with the corresponding primary and secondary antibodies, and the protein signal was detected using the chemiluminescence imager Tanon 5200 (Tanon Co., Ltd., Shanghai, China). The antibodies, dilution factors, sources and other information are presented in Table 1. The protein band gray value was analyzed using ImageJ software. The relative expression levels were calculated by comparing them to the expression of the β-actin.

### 4.8. Statistical Analysis

Statistical analysis of all data was conducted using GraphPad Prism version 8.0 software. All results are expressed as the Mean ± SD. Statistical significance was determined through one-way ANOVA or unpaired Student’s *t*-test by using Tukey’s post hoc test. *p* values of less than 0.05 were considered statistically significant. The software showed a normal distribution.

## 5. Conclusions

In conclusion, the current study demonstrated the reno-protective effects of paricalcitol against cisplatin-induced acute kidney injury in mice. Mechanistically, the renal protective effect of paricalcitol is related to the activation of the *Nrf2*/*HO-1* signaling pathway, which is associated with anti-oxidant and anti-inflammatory effects. Our study shows the preventive effect of paricalcitol on cisplatin-induced acute kidney injury, which not only provides more experimental data for the pharmacological effects of paricalcitol, but also provides a new idea for the clinical prevention of drug nephrotoxicity.

## Figures and Tables

**Table 1:** Antibody dilution.

Antibody	Dilution Ratio	Manufacturer	Molecular Weight
β-actin	1:500	Wanlei	42 KDa
Cas8	1:1000	CSA	43 KDa
PARP1	1:500	Proteintech	89 KDa
IL-1β	1:1000	Wanlei	31/35 KDa
TNF-α	1:500	Wanlei	26 KDa
NF-κB	1:500	Wanlei	65 KDa

## Abbreviations

BUN	Blood urea nitrogen
Scr	Serum creatinine
ROS	Reactive oxygen species
MDA	Malondialdehyde
SOD	Superoxide dismutase
GPX4	Glutathione peroxidase 4
GSH	Glutathione
T-AOC	Total antioxidant capacity
RIP1	Receptor interacting protein kinase 1
RIP3	Receptor interacting protein kinase 3
Cas8	Cysteinyl aspartate specific proteinase 8
PARP-1	Polymerase-1
TNF-α	Tumor necrosis factor-α
NF-κB	Nuclear factor kappa B
IL-1β	Interleukin-1β
Nrf2	Nuclear factor erythroid 2-related factor 2
HO-1	Heme oxygenases 1
H&E	Hematoxylin-eosin staining
TEM	Transmission electron microscope
IF	Immunofluorescence
